# “Will You Still Feel Beautiful When You Find Out You Are Different?”: Parents’ Experiences, Reflections, and Appearance-Focused Conversations About Their Child’s Visible Difference

**DOI:** 10.1177/10497323211039205

**Published:** 2021-10-01

**Authors:** Kristin J. Billaud Feragen, Anita Myhre, Nicola Marie Stock

**Affiliations:** 1 Oslo University Hospital, Oslo, Norway; 2 University of the West of England, Bristol, Bristol, United Kingdom

**Keywords:** visible difference, appearance, craniofacial, qualitative, parent, Thematic analysis, appearance-altering surgery, Europe/Norway

## Abstract

To investigate parents’ reflections and experiences of having a child born with an appearance-altering condition, interviews with 33 parents of children born with rare craniofacial conditions were analyzed using inductive thematic analysis. Three themes emerged: “Managing emotions: A dynamic process,” “Through another lens: External reminders of difference,” and “Awareness of difference: Approaching the child.” Findings suggest that although parents learned to accept and love their child’s visible difference, external factors such as appearance-altering surgery and other people’s reactions activated difficult emotions in parents. Parents struggled to decipher whether and when to raise appearance-related issues with their child, and how this could be done without distressing the child. Anticipatory guidance that facilitates positive appearance-focused conversations both within and outside the home seems to be needed. Parenting skills could also be strengthened by preparing parents for social reactions to the child’s visible difference, and their child’s changed appearance following surgery.

## Introduction

While appearance concerns are now considered “normative” within the general population (Harcourt & Rumsey, 2012; Tantleff-Dunn et al., 2011), those born with an appearance-altering condition (“visible difference”) vary not only from the societal appearance ideal, but also from the norm. A congenital craniofacial anomaly (CFA) is a broad term used to describe a wide range of conditions that affect the appearance and function of the head and face (Buchanan et al., 2014). Depending on the severity of the condition, complex multidisciplinary treatment to “correct” the anomaly may be required throughout childhood and often into adulthood. In spite of surgical and other interventional procedures from birth to adulthood, affected individuals may feel that they differ from their peers in terms of facial appearance (Beaune et al., 2004; Feragen & Stock, 2017). Some craniofacial conditions, such as Apert syndrome, also visibly affect hands and feet (Buchanan et al., 2014). Strangers and/or peers may react to differences in the child’s appearance, leading to unwanted staring, questions, comments, and teasing, as has been extensively demonstrated by research on visible differences in general (Rumsey & Harcourt, 2004; Uttjek et al., 2007), and craniofacial conditions in particular (Boltshauser et al., 2003; Fischer et al., 2014; Roberts & Shute, 2011).

For parents, feelings of shock, grief, anger, guilt, and/or anxiety about the child’s future are common following their child’s diagnosis (Klein et al., 2006; A. M. Nelson, 2002; P. A. Nelson, Glenny, et al., 2012; Pope et al., 2005). Parents must support their children through a series of surgical interventions (Feragen et al., 2019), many of which will change their child’s appearance. Although previous research has found parents to be satisfied with the outcomes of their children’s appearance following surgery (Feragen & Stock, 2017), parents must nonetheless face what is experienced as the challenging task of making difficult decisions on their child’s behalf (van Manen, 2014). Parents of children with cleft lip and palate also often refer to an underlying threat of negative social experiences and worry that appearance-related comments and behaviors from others may lead to their child feeling less socially accepted (P. A. Nelson, Glenny, et al., 2012), which parents of children with craniofacial conditions fear could lead to social withdrawal (Sarimski, 2001). In the longer term, parents’ emotional reactions to their child’s visible difference, the ongoing burden of treatment, and the experience and/or fear of negative social reactions can impact parents’ overall quality of life and overall levels of stress (Bannink et al., 2011; J. M. Rosenberg et al., 2011).

While research has described the broad psychological impact of having a child born with a visible difference such as a craniofacial condition, very few studies have specifically focused on how parents perceive their child’s different appearance, or how they choose to address this complex and possibly sensitive issue with their child (Zelihić et al., 2021). There is also little knowledge about facilitators, barriers, and challenges experienced by families when choosing to communicate, or not, about their child’s medical condition (O’Toole et al., 2015). Furthermore, qualitative research is lacking in the craniofacial field (Feragen & Stock, 2017), despite its ability to provide unique and detailed insight into parents’ experiences, allowing researchers to uncover meaning and gain understanding (Murray & Chamberlain, 1998; P. A. Nelson, 2009). To help parents to cope with their child’s ongoing medical care and the everyday challenges of social visibility, health professionals need to understand parents’ key stressors and experiences. Utilizing a sample of parents of children born with rare craniofacial conditions, the aim of the present study was to explore parents’ experiences and reflections about their child’s appearance and treatment, using an in-depth inductive qualitative approach.

## Method

### Design

Qualitative data were collected as part of a larger study, with the overall aim of strengthening our understanding of treatment experiences in young people and adults with a rare craniofacial condition and their parents, and the same participants’ thoughts and experiences regarding growing up with a congenital and visible facial difference (Myhre et al., 2019). For the full study, a semi-structured interview guide was created by the authors by drawing upon the first and third authors’ research knowledge from the craniofacial and broader health fields, in addition to the first author’s clinical experience with this patient group. The semi-structured nature of the interview ensured that pertinent issues would be investigated, while also allowing participants to share their own narrative of the experiences they considered most relevant. Participants were asked open-ended questions, and were prompted to provide more details where appropriate. The full interviews from the larger study consisted of two separate parts. The first part explored parents’ experiences with treatment and with specialized health professionals; more specifically their perceptions of the information provided about the child’s condition and its treatment, experiences of shared treatment decision-making, and the quality of communication with health professionals. The second part of the interview investigated issues related to the child’s appearance and social experiences, seen from the parents’ perspective. Data related to the second part of the interviews were extracted from the transcripts. In addition, if parents talked about their child’s appearance when asked about their child’s diagnosis in the first part of the interview, such data were also extracted and added to the material, and analyzed for the purpose of the present study. This second part of the interview guide, along with exemplar questions, can be found in the Supplemental file.

### Research Team

The research team consisted of three researchers. The first author is a qualified clinical psychologist with a PhD, and more than 20 years of research and clinical experience in appearance psychology and craniofacial conditions. The second author is also a clinical psychologist and a PhD candidate, currently working on a thesis focusing on adjustment to a craniofacial condition in parents and adults. The third author has a master in health psychology and psychological methods, and 10 years of research experience on craniofacial conditions. Interviews were performed by the second author. At the time of the interviews, the second author had limited research and clinical experience with the craniofacial population and is not a member of the multidisciplinary treatment team in charge of the patients’ follow-up and treatment. The first and second author have clinical experience with treating individuals born with a craniofacial condition, but no participants had any therapeutic relationship with the authors prior to the interviews. All authors were female and none have any direct personal experience with living with a visible difference.

### Procedure

Centralized multidisciplinary care for the treatment and follow-up of all patients born with a CFA is provided in Norway. Patients receive regular invitations from The National Unit for Craniofacial Surgery to attend a multidisciplinary consultation, the frequency of which depends on the complexity of the condition and individual need for follow-up. All parents attending a multidisciplinary consultation with the craniofacial team from September 2016 to October 2017 were invited to participate in an interview about their experiences. Information about the study and a consent form were sent to parents by post prior to the consultation, with details about what participation in the study would entail, and key ethical information such as confidentiality and right to withdraw. After participants had returned the consent form by post, an appointment for the interview was made over the telephone. Those who did not contact the researchers prior to the multidisciplinary consultation received written information about the study at the clinic.

### Participants

Given the heterogeneity of rare craniofacial conditions, a large sample was collected, to ensure saturation of parental experiences across conditions with differing severity. Hence, a total of 81 parents who attended a multidisciplinary craniofacial consultation were informed about the study. Five (6.5%) chose not to participate. Fourteen parents (17%) responded positively, but subsequently were not reached when contacted for an interview appointment. A further 25% (*n* = 20) did not respond, which could indicate a lack of available time, a lack of felt relevance or wish to participate, or the absence of up-to-date contact information. In the first phase of the study, six additional parents contacted the research team directly, wishing to participate. Forty-eight parents provided informed consent and were successfully reached for an interview.

To reduce the heterogeneity of the sample, and include parents for whom the research questions would be most relevant, data from some participants were subsequently excluded from the present study. Inclusion criteria were that the condition would affect the child’s face and head, and treatment could be offered to “correct” the visible difference, in addition to functional repair. Data for 15 parents were therefore excluded from the current study based on the following criteria: (a) the child’s craniofacial condition was degenerative, introducing an additional stressor in parents and different treatment approaches and outcomes (*n* = 4), (b) the condition did not significantly affect the child’s current appearance and surgical interventions had happened only once and soon after birth (single suture craniosynostoses or conditions not primarily affecting the head and face, *n* = 10), or (c) the child had very recently been adopted (*n* = 1), had not yet received treatment from the multidisciplinary team, that parents’ experiences were still primarily focussed on the child’s adjustment to his/her new family and environment.

In total, 33 parents of 32 children contributed interview data to the present study (6 fathers, 27 mothers). Participants’ children were aged 1 to 18 years (mean age = 8.8 years). Ten children were female while 23 were male. Parents of very young children were not interviewed about how they talked with their children about appearance, but were invited to share their thoughts about doing this in the future. Children’s conditions included Treacher Collins, Crouzon, Goldenhar, Muenke, and Apert syndromes, in addition to some other very rare genetic conditions that will not be named in order to protect participants’ anonymity. Two parents had a craniofacial condition themselves. Two children had an additional diagnosis of autism spectrum disorder and/or severe cognitive difficulties.

Interviews were audio-recorded with participants’ permission and according to the participant’s preference were conducted face-to-face (*n* = 22) or over the telephone (*n* = 11) Interviews for the original study, from which the data for the present study were extracted, lasted 60 minutes on average (range 30–75 minutes).

### Analysis

Thematic analysis is ideal for identifying, analyzing, and presenting broad patterns or themes within qualitative data sets and was mainly carried out by the first and third authors following the guidance and six step protocol provided by Braun and Clarke (2006).

Interviews were transcribed verbatim. All authors read all interviews several times, to become familiar with the data, and identify interesting features. During this process, the first and third author discussed the potential of investigating parents’ perceptions of their child’s appearance and treatment by exploring this specific issue in the data set. The relevant sections of the interviews were subsequently extracted from the transcripts and translated from Norwegian into English. The analysis was inductively driven, so that coding and theme development were grounded in the data. Simple coding was first performed by hand on the printed interviews, and subsequently transferred to an Excel-document, where consensus coding was performed by the first and third authors. The first and third authors then organized the codings into themes, based on their emergence across cases and their explanatory value within cases, and all three authors reviewed the themes identified as central. Themes were chosen for their prevalence and/or their importance in relation to the research questions and were cross-checked and discussed until full agreement was reached. Last, themes were defined and named, and structured according to their content. Thematic maps were used to organize and re-organize the structure between and within key themes. The organization of themes was seen as a recursive process, involving all three authors, and several attempts to organize themes diligently were carried out, before the current structure was adopted.

As suggested by Hill et al. (2005), the representativeness of the present study’s results across participants is described by categorizing findings as general (applied to all or all but one case), typical (more than half of cases), or showing variance (less than half but more than two cases). When reporting the findings, these three labels are referred to as all (general), most (typical), and some (variance).

### Ethical Considerations

The Data Protection Office at Oslo University Hospital granted ethical approval for the study (2016/14088). The interviewer assured participants of confidentiality of all provided information. If needed, referrals or a subsequent follow-up could be arranged by the clinical psychologist performing the interviews.

## Findings

Three key themes were identified: (1) Managing emotions: A dynamic process; (2) Through another lens: External reminders of difference; and (3) Awareness of difference: Approaching the child. Each theme and corresponding subthemes are described below in more detail and supported by direct exemplar quotes from participants. Square brackets [ ] are used to indicate author’s clarifications, such as when participants refer to previous information or use pronouns. Parentheses (. . .) indicate that some extract has been removed for editorial concision, for example, ineffectual text (such as “umm”) or irrelevant text (associations or repetitions).

### Theme 1—Managing Emotions: A Dynamic Process

The first theme describes a range of different and often challenging emotional reactions experienced by parents in relation to their child’s visible difference. These emotions seemed to originate from personal, internal processes, but were also closely intertwined with social experiences, anticipated future experiences, and the experience of the child’s changed appearance following surgery.

#### Emotional reactions to the visible difference

Most parents described a range of difficult feelings upon learning that their child had a visible difference. For example, one mother described her experience as “kind of disturbing” and felt it was “easier” to look at the unaffected side of her daughter’s face. Some parents mentioned it felt particularly challenging that the condition affected the child’s face: “We felt discomfort over this difference and that the difference was in her face, since this is the first thing you see.” The reason for this seemed to be closely related to other people’s potential reactions and attitudes. Parents feared that the child’s difference would shape other people’s “first impressions—you don’t want it to be like that.”

Over time, most parents described a process of acceptance: “In the beginning, I didn’t like it,” but *“you learn to love the difference.”* However, the pathway to acceptance had been challenging for a few. One mother shared that for weeks after the child’s birth, they did not show their child to others, not even to their family, because of a need to “feel ready ourselves first and be used to him.”

Another interesting finding was what some parents described as the unexpected lack of resemblance to other family members, combined with a similarity to “other [children] with the same syndrome.” This generated sadness or sorrow, and a need to get used to not recognizing the child’s features as similar to other family members:

In the beginning, it was so different. I thought he looked really strange and it took a while before I managed to adjust to his appearance, to get used to him; I think it was a natural process, because we had imagined he would look like us and his siblings, but he didn’t.

When asked about how they felt about the child’s appearance, most parents provided descriptions of their child’s physical features, or responded that they were used to the child’s appearance and therefore did not think much about it anymore. In contrast, one father described how negative feelings about his child’s appearance led to feelings of shame:

I had a period where I felt [the difference] was a little disgusting, or some kind of shameful feeling, and you could realise you had the thought “should I take him out with me or not?” and then you felt ashamed of that feeling too.

#### Emotional reactions to other people’s responses

Social reactions could be experienced as harsh or bewildering. When confronted with other people’s unexpected behaviors, some parents shared how they had been caught off-guard, and as a consequence, had not managed to respond in the way they would have liked: “It just appears and you become completely paralysed and can’t say a word.” Social reactions had hurt their feelings on the child’s behalf, led to sadness or irritation, and activated a need to protect their child from other people’s responses to the visible difference.

The interviews also illustrated how some parents worried for their child’s future because of their anticipation of other people’s reactions to the visible difference. As an example, one mother shared her first thought after the child’s birth: “What would happen when he went to college (. . .), where it is all about appearance?” Other parents reflected more generally on the role that appearance plays in our society, “it’s all about looking perfect (. . .), looking normal (. . .). We are not within the norm at all.”

In addition to overtly confrontational social experiences, some parents also observed an absence of positive reactions that could be experienced as hurtful or lead to sadness or loss.

One of our first experiences was at the hospital after birth (. . .), with the baby in its cot (. . .), and there might be some grandparents in the elevator, saying “awwwww” [at other babies], but we never had that.

As mentioned above, some parents needed time to get used to the child’s appearance and described this as a process that had strengthened their love for the child and increased their social confidence. Parents who felt secure and confident about their child’s appearance felt it was easier to cope with challenging social experiences, even when interactions were experienced as hurtful or difficult: “It is easier now to cope (. . .) I have gotten used to the situation and I feel more safe (. . .), he is my son however he looks.” Acceptance of their child, by themselves or others, seemed to be the end point of this process:

I went through a process and when I landed, I found out my son is my son, and I will fight for him to be able to be himself always.

#### Emotional reactions to a change in appearance after surgery

Parents shared several ambivalent feelings triggered by thoughts about or experiences of their child undergoing appearance-altering surgery. For parents struggling to accept their child’s different appearance, surgery could be seen as a welcome solution for the distress they were experiencing, conveying hopes that the child’s appearance would “become more normalised.” Some parents pre-empted difficult social situations and wanted the visible difference “to be repaired as quickly as possible (. . .), because children bully.” One family had “pushed the surgeons” to perform the operations before Kindergarten, to “protect ourselves as parents” and shield the child from questions and comments.

Still, approximately half of the parents found it extremely demanding to expose their child to post-surgical pain and/or change their appearance. Difficult emotions included mourning, sadness, and/or feeling conflicted about what was best, irrespective of whether surgery was performed on the child’s head, face, or hands. Some feared that agreeing to surgery would convey a message of non-acceptance of the visible difference to their child, which again led to emotional ambivalence:

The surgery gnawed away at me. We had to decide for him that he was not pretty enough. That was really not OK, because I didn’t want to say “you look like we need to fix you.”

Appearance-altering surgery activated new thoughts and feelings, and a need to process the change. A change in their child’s appearance could be experienced as reinstating normalcy but could also feel confusing:

You don’t love him less because he is different and you learn to love the difference, so I felt it was sad when his hands were operated on (. . .), but of course we can see that operating is a good thing.

Post-operative appearance (bruising and swelling) and/or permanent changes to the child’s appearance as a result of surgery could also feel confusing or strange and took time for parents to adjust to. A father described how his attempt to visualize how his son would look after surgery was disturbed by his son’s face being “swollen and bruised,” knowing it would take “weeks and sometimes months before [his appearance] stabilised.” One mother recalled the puzzling experience it had been that her son “smelled and sounded” as she remembered, yet she “didn’t recognise him” when she saw him for the first time after surgery.

Some parents described how surgery had helped them to see the child they had originally imagined, “The operation gave him a more whole face. This is how he should have looked,” in some cases leading to overwhelmingly positive feelings as the result of the outcome of surgery:

My God, after the first operation I cried so much from joy, and I gave the surgeon a hug for the job he’d done, it was incredible, we were so happy, I will never forget that day.

Some parents also described how operations had restored a family resemblance:

She opened her eyes and suddenly, she looked like her father so much (. . .). It was a fantastic feeling.

In one way or another, appearance-altering surgery had a powerful impact on some parents’ perceptions of the child’s identity as theirs and/or as different. All parents found the experience to be emotionally demanding:

We came into the [post-operating] room (. . .), there were many children there, and it took some time for me to see which one was mine. (. . .) [But then my daughter] cried and my mother’s instinct just took over. My husband started crying (. . .), he needed a minute to cope with his feelings (. . .). [It was strange] going home with a child that looked so different, but also a big relief, she looked so nice and it had gone so well.

### Theme 2—Through Another Lens: External Reminders of Difference

The second theme illustrates how parents are reminded of the visible difference that has become invisible to most parents on a daily basis. Photographs and mirrors were defined as often surprising external reminders of the visible condition, in addition to the many social situations generated by the visibility of their child’s condition.

#### What other people see

Most parents could not see the child’s difference after a while, “I have other eyes, so I can’t really assess if she is different,” but remained acutely aware of others noticing and described how social encounters became a reminder of the child’s different appearance. In most cases, reactions were characterized by non-judgmental surprise or curiosity, but some parents also observed and/or feared other people’s assumptions about their child’s abilities and health. One mother wondered whether social curiosity was restricted by a “social norm,” where enquiring about the cause of the visible difference was less acceptable if perceived as being congenital, and therefore often not addressed. This was contrasted by the relative safety of mentioning the difference if it was assumed to be a result of an accident:

If [my son] was not wearing a hat, nobody talked to us, since they could see [he had] a syndrome (. . .). When he was wearing a hat (. . .) and he had a lot of bandages on his hands after his [hand] operation, people (. . .) asked “oh, poor thing, what has happened, was it an accident?” (. . .) These questions are acceptable, people do not find that problematic at all, but if it is congenital and different, then you can’t ask.

Parents shared experiences of people staring or “pretending they do not see” when they took their child out in public. Parents found children’s “pointing and whispering” understandable, but found adults’ behaviors more difficult to process, reflecting on how “few manners some people have”:

I couldn’t go out without people turning around and staring and looking shocked (. . .). I’m not exaggerating, [I’ve had] people hanging over the [shop] counter and saying ‘oh wow, I haven’t seen this before’ (. . .). You don’t point at people and say things (. . .) and I thought it was strange that grown up adults would do that.

Having had difficult social experiences, some parents wished others could choose to see their child’s other features, such as “the nice eyes, beautiful smile, nice dress, and the wonderful human being that is there,” instead of focusing on the difference, probably mirroring their own perception of their child. Correspondingly, participants hoped other parents could help their own curious children to respond and behave in a more helpful way:

Other parents not daring to ask, hushing their children (. . .). It would be better if they let their child ask, because when they say ‘hush, he is different, don’t look at him,’ then (. . .) he’s excluded, he’s categorised as strange.

In response to difficult social experiences, some parents wanted to improve other people’s understanding of their child’s visible difference. By providing information to their child’s Kindergarten or school, they believed they could “make [the difference] less threatening,” which would in turn create a safer environment for their child. Parents who had prepared others in this way described how they believed this action had reduced a potentially negative impact of the difference:

Before he started [school](. . .), we made a PowerPoint presentation that was shown to all classes (. . .). The PowerPoint had pictures of my son as a baby and you could see how different he was and all the operations he’d had. We tried to make it so they could feel empathy and understand what he’d been through. (. . .) On his first school day, we heard lots of kids saying hello to him (. . .). He was so happy, and everyone had the information they needed.

In contrast, one father feared that sharing information about his daughter’s condition would single her out in a negative way, leading to stigmatization, or activating difficult feelings in the child:

One has to be careful about the words one uses (. . .). It can be hurtful, not only for children, but also for parents, to be confronted with [the difference]. We want [our daughter] to be treated the same, so we believe we shouldn’t focus on [the difference].

#### The power of reflected images

Additional external reminders of the child’s visible difference were identified in the form of mirrors and photographs. When looking at photographs, some parents had been surprised by the difference between “him in movement and him in a photo. You see [the difference] really well when you don’t have that 3D effect.” Another mother commented,

Her smile and facial expressions: that is my child. [But] as soon as I see a photograph, I see how dramatic [the difference] is.

Mirror reflections were identified as particularly surprising in their ability to reveal or enhance the child’s visible difference. Several parents described how they had been startled by the reversed image of their child, and some described hurt or sadness following this unexpected reminder:

One reacts differently when one sees it in the mirror because there’s another dimension there (. . .). I thought that was really hard in the beginning. It becomes very clear that [my child’s face] is not symmetrical (. . .). That was really difficult for me to accept (. . .). It was like looking at someone else, suddenly I saw something different and I wasn’t prepared.

One father also explained how coming home after being away for some time enhanced his perception of the visible difference, “wow, you really look different.” Parents also described how seeing their child with “other children their age” increased the “contrast” and served as a reminder of their child’s visible difference.

### Theme 3—Awareness of Difference: Approaching the Child

The third theme describes parents’ thoughts, feelings, reflections, and dilemmas regarding whether, when, and how to talk about the different appearance with their child. This theme also includes parents’ attempts to guess the child’s feelings about their visible difference, which shaped parents’ reflections about whether or not to address the issue.

#### Silent observations

Most parents seemed to base their understanding of their child’s self-awareness on observations and assumptions rather than conversations, using words such as “I think,” “I believe,” or “we haven’t heard her comment on her appearance.” The interviews provide many examples of parents silently observing their child’s curiosity or discovery of their visible condition, and most parents knew whether their child was aware of having a visible difference or not. Few, however, knew how the child felt about this. Most parents seemed to prefer to observe their child’s behavior without interfering: “I saw him in the bathroom looking in the mirror and he was turning his head in different directions.”

#### Initial questions about the difference

The child’s growing consciousness about their visible difference could be followed by the child asking the parents an appearance-related question, as described by one mother:

Not long ago she stood in front of the mirror and she was looking for a long time, and then she asked, “what is it I have on my face?” This is the first time she has asked about her scar, so that was an interesting moment, knowing that she now wonders what this is.

If the child had raised questions regarding his or her different appearance, parents seemed comfortable enough to describe the condition’s physical characteristics to their child. When doing so, parents also gave “positive feedback” about the child’s characteristics “not just related to appearance, but generally.” Parents also underlined the unique individual appearance in an attempt to counteract a potentially negative impact of the visible difference on their child’s self-confidence, by telling the child that “we are all created differently, everyone has a different face.”

To help their child understand the features of their condition and the treatment they had been through, several parents had collected photographs and texts, with the intention of showing them to their child and using them when talking about the visible difference. Parents described the importance of their child being able to follow their treatment pathway and to “read about it when he is older.” Photographs and treatment narratives were mentioned by some parents as a helpful tool if their child had asked questions about their different appearance, and in anticipation of future conversations.

#### Taking about the difference: Reflections and fears

Having observed their child’s growing awareness of their visible difference, some parents reflected upon whether this could present an opportunity for them to initiate a conversation about appearance with their child: “It could have been that someone made a comment, and I thought afterwards that I should have asked her.” One mother recounted having tried to start the conversation, but felt their child had not been interested. Still, few parents had actually found the right opportunity to raise the issue or felt confident enough to do so, but believed that raising the issue of appearance in advance of social reactions or questions would be ideal, as described by one father:

He is starting school [soon] and suddenly [the difference] will become clear. It’s a new, big environment with lots of new kids and probably there will be some attention, especially in the beginning, so we will talk with him in advance.

Almost all parents struggled with doubts about whether to take the initiative to explore their child’s emotions about having a different appearance, describing it as one of the fathers as a “double-edged sword.” Parents questioned how to initiate an appearance-focused conversation without generating insecurities in the child and described how they were afraid of creating a negative awareness, choosing the wrong moment, or distressing the child by raising a sensitive issue. Parents knew this conversation would at some point become unavoidable, but not knowing when and how to address it led to conflicting emotions, engrained in a fear of generating appearance-related distress:

Should I discuss it with her before she finds out by herself? Some say you should [but](. . .) I don’t want to create a problem for her, [making her feel]: “are you aware of being different?” We feel really unsure about how to deal with this (. . .). I may have to ask her, but I feel it’s a hurdle. How will this talk affect her?

As an alternative strategy, some parents felt it was better just to make room for talking about the difference if the child chose to raise it themselves. Others worked on normalizing appearance, hoping that such conversations could help the child to feel confident to raise the issue when they were ready.

This is a balance between normalising and not having too much focus on it, but at the same time something is different and it’s important to make it possible for her to wonder or ask questions so that we can help her (. . .). I need to trust that she tells me what she thinks about her own appearance.

## Discussion

The aim of this study was to explore parents’ experiences and reflections in relation to their child’s different appearance using an in-depth inductive qualitative approach. The results illustrate a pathway of acceptance, intertwined with a range of challenging emotions related to specific settings or external reminders, such as appearance-altering surgery or other people’s comments or behaviors. Most parents struggled to decipher whether to talk about appearance with their child and when, and how to have these conversations without causing distress. Findings shed light on the everyday challenges faced by parents of children with a visible difference and have implications for future research and the clinical management of appearance-related issues.

### When External Reminders Hurt

Congenital craniofacial conditions are characterized by a myriad of features that may be experienced as different (Rumsey & Harcourt, 2004). When asked how they felt about their child’s appearance, most parents provided factual physical descriptions of their child’s difference, and responded that they were used to their child’s appearance and didn’t think about it, unless they were reminded of it. Most parents said that they had accepted their child’s condition and learned to love the visible difference. They also found other people’s reactions hurtful and felt a strong need to protect their child, in line with research on other conditions with a socially visible component (Currie & Szabo, 2020). Previous research has also underlined that regardless of how well individuals may adjust to a specific condition, social situations nevertheless continue to act as reminders of individuals’ and society’s attitudes toward visible difference (Konradsen et al., 2012; Vehkakoski, 2007).

Parents described a range of emotions when talking about the child’s visible condition, such as fear for the child’s future, sadness, and in some cases discomfort or shame. Over time, research has shown that strong emotions may dissipate, as parents accept the situation and focus on the child’s needs (Knafl & Gilliss, 2002; Smith et al., 2015). However, when parenting a child with a visible condition, external social factors may complicate the process of adjustment, as illustrated in the present study across all themes. Sadness and fear could be associated with the anticipation of other people’s reactions, judgments, and negative assumptions, in line with previous research (Currie & Szabo, 2020; Frances, 2004; Klein et al., 2006, 2010, 2014; Pope et al., 2005; Roberts & Shute, 2011). Other people’s reactions were described as challenging, disturbing, and/or distressing, such as when strangers made inaccurate assumptions, kept their own children away, or made insensitive comments. Some parents also described feeling invisible in situations when strangers would normally initiate a conversation or make a positive comment, previously described as silent language or silencing conditions (Konradsen et al., 2012). In such situations, parents may feel their child is “set apart” or not “belonging,” which can activate feelings of rejection or social exclusion, and trigger powerful emotions (Molden et al., 2009; Uttjek et al., 2007). According to Molden et al. (2009), belonging and connectedness may be threatened by other people’s explicit and active, or implicit and passive behaviors. Social reactions to the visible difference could therefore, to varying degrees, represent or be perceived as threats to the desire to be accepted and appreciated by others (Smart Richman & Leary, 2009), which explains the emotional impact of such experiences. While the anticipation and occurrence of explicit social reactions to a visible difference has been well documented within the craniofacial literature (Feragen & Stock, 2017), passive and implicit indications of social disconnection are less investigated, despite this being recognized as a highly emotionally damaging form of social exchange (Saylor et al., 2013). The ability to cope with experienced or feared loss of social belongingness is central to psychological well-being (Smart Richman & Leary, 2009). To better support parents of children with a visible condition, clinicians need to understand the many factors associated with the potential stress of social reactions and be able to identify those who struggle.

As a way of protecting their child from hurtful or insensitive social reactions, and in an attempt to build their child’s self-confidence, parents described a range of strategies, choices, and behaviors, some of which have been described previously in relation to visible conditions (Franzblau et al., 2015; Klein et al., 2006, 2014; Uttjek et al., 2007). Examples included informing other children and adults about the condition, its consequences and treatment, so that others would be prepared. Studies have demonstrated that informing others about the condition can not only improve other’s understanding and empathy, but can help parents and affected children to regain control of the situation, and enhance self-management (Frances, 2004; Jackson et al., 2012; Schulman-Green et al., 2016). Yet, a few parents preferred not to inform others, fearing that the attention given to the visible difference would lead to stigmatization or highlight the difference in a negative way. More knowledge about advantages and disadvantages of sharing information about a child’s condition and its potential correlates to, for example, kinder gardens and schools is needed.

### Talking About Appearance: A “Double-Edged Sword”

Research on children with medical conditions has demonstrated the importance and health-related gains of open parent–child communication about a medical condition (Middleton et al., 2018; O’Toole et al., 2021), more specifically when the child is overweight (see Gillison et al., 2016), in cases of trauma (McGuire et al., 2019), and in terminal cancer (see A. R. Rosenberg et al., 2016). These studies provide growing evidence that sensitive, timely, and age-appropriate information is helpful and important for a child, even in cases of distressful disclosures (Aldridge et al., 2017). Research also indicates that helping parents with this delicate task may also have the potential to strengthen their perception of self-efficacy, to the benefit of both parents and the child (Albanese et al., 2018).

In spite of evidence of the positive impact of such conversations, there is little knowledge about factors that may facilitate or complicate communication about a child’s medical condition within the family (O’Toole et al., 2015). To the authors’ knowledge, very few studies have investigated how parents of children with craniofacial conditions experience talking with their child about the visible difference, and how health professionals involved in their care could support parents in this sensitive task. One recent study, including young people with a range of different visible conditions, has confirmed that parents find the issue of a different appearance difficult to raise with their child, mostly because they are afraid of creating or strengthening appearance distress in their child by talking about the difference in appearance (Zelihić et al., 2021). This study also showed that parents’ emotional reactions to the visible difference, if experienced as difficult, complicated the initiation of a conversation about appearance (Zelihić et al., 2021). Research has demonstrated that parental distress has the potential to impact considerably on the child’s own emotional development (e.g., Pope et al., 2005). It is therefore likely that parents who have managed to adjust positively to their child’s condition will be better equipped to help their child to develop a positive and strong self-image and also be more emotionally prepared for an open-minded conversation with their child about the difference in appearance. This highlights the need for health professionals to support parents in coping with potentially challenging emotions related to their child’s visible condition, hereby strengthening their ability to meet their child’s needs.

Our participants felt it was difficult to discuss appearance issues with their child, unless conversations were factual talks offering descriptions of the physical difference. However, parents did make a conscious effort to build their child’s self-confidence by underlining individual uniqueness and strengthening their child’s awareness of their other attractive characteristics and/or features, as demonstrated in other studies (Franzblau et al., 2015; A. M. Nelson, 2002). Nonetheless, parents remained reluctant to bring the issue forward, even when some children had asked questions about their appearance, fearing they would generate insecurities or engender appearance concerns, as also demonstrated in a recent study on parents of children with a visible difference (Feragen et al., 2019), and in other medical conditions (Middleton et al., 2018; O’Toole et al., 2021). Self-doubt has been described as prevalent in mothers of children with disabilities (A. M. Nelson, 2002), an understandable and normal reaction to unusual circumstances. Most parents expressed insecurity as to whether, how, and when initiate a conversation about the child’s different appearance. The dilemma of deciding whether or not to take the initiative to talk to the child about appearance was described as a double edged-sword and an internal struggle: the ultimately unavoidable nature of this conversation combined with the fear of its emotional consequences for the child. Some parents may feel trapped between their child’s right to know and their desire to protect him or her from information they fear could be difficult for their child to process. The present study seems to provide evidence that parents see the value of parent–child conversations about the visible difference, but also suggests that some parents would benefit from additional support to have the courage to do so, feel competent while doing it, and manage to tailor the conversation to the child’s needs and developmental stage. Given that communication is known to promote acceptance and adjustment to a health condition, and mediate child health outcomes (Middleton et al., 2018), strengthening condition-specific communication skills and guiding parents in their struggle to find the right moment to address appearance issues with their child with sensitivity, seems essential for the child’s psychological health and development (Aldridge et al., 2017).

### A Change in Appearance: Surgical Interventions

Parents’ experiences of their child’s appearance-altering surgery triggered elaborate reflections and emotions, ranging from happiness and relief at the success of the surgery, to mourning and sadness over having lost their child’s pre-surgical appearance. Following surgery, some parents were emotionally overwhelmed by not being able to recognize their child, either because of post-surgical bruising and swelling, and/or related to a changed appearance. Some parents explicitly mentioned how the operation had restored a family resemblance and/or were grateful that the surgery had had a normalizing effect, underlining the social importance of normalcy, as exemplified by a review of qualitative studies (A. M. Nelson, 2002).

As identified in previous research (Bull & Grogan, 2010; P. A. Nelson, Caress, et al., 2012), some parents experienced conflicting emotions around having sanctioned their child’s appearance-altering surgery and felt guilty for putting their child in a potentially painful situation, which they also feared could convey a message to their child of non-acceptance of the visible difference. The approval of surgery could also be experienced as threatening the parents’ role as the child’s protector, since parents could not protect their child from pain (Bull & Grogan, 2010). Agreeing to their child undergoing surgical intervention has been described as implying feelings of weight, burden, and charge, but also perceived benefits for the child in the longer term (van Manen, 2014). This ethical dilemma could explain why parents seemed trapped between their adjustment to their child’s different appearance and their experience or anticipation of other people’s negative reactions. Surgery could therefore be seen as a protection against other people’s lack of acceptance. Hence, results from the present study could illustrate what has been described in previous research as the “embrace of paradox”: parents’ acceptance of the child as it is, contrasted with never giving up hope for improvement or possible change (A. M. Nelson, 2002).

Parents need to be prepared for changes in their child’s appearance following surgery, as well as for possible emotional reactions to this change. Individualized support and information can empower parents to fulfill their parental role with more confidence and reduce feelings of helplessness (Bull & Grogan, 2010). In parallel, unrealistic expectations of treatment outcomes have been shown to be associated with parental distress (Rumsey & Harcourt, 2004; Williamson & Rumsey, 2017), underlining the importance of communication in health care settings. Parents should be informed about post-surgical bruising and swelling, how long recovery could be expected to take, as well as longer term changes in appearance. Information should also include the normalization of strong and conflicting emotions surrounding appearance-altering surgery.

### Strengths and Limitations

Few studies have explicitly and exclusively focused on how parents experience their child’s different appearance, and how they experience talking to their child about the difference. The themes derived from the present study could therefore be useful in helping clinicians and researchers to better understand parental perspectives following the birth of a child a visible condition. The study’s inductive qualitative approach and relatively large sample drawn from a centralized treatment setting is a strength, particularly in the context of few existing qualitative studies on rare craniofacial conditions (Feragen & Stock, 2017). Interviews were performed by the same author, reducing the possibility for differences in interviewer technique and characteristics. Interviews were performed either face-to-face or by telephone, in line with research supporting the importance of giving participants the opportunity to choose the method they feel most comfortable with, particularly when the topic of interest is sensitive in nature (Heath et al., 2018). A final strength was the inclusion of fathers, since fathers’ views and experiences are largely missing from the craniofacial literature (Klein et al., 2010). Nevertheless, the number of fathers was still restricted, and future research should aim to appeal to a higher number of fathers.

Two of the children in the sample had an additional diagnosis of autism spectrum disorder and/or severe cognitive difficulties. The presence of developmental difficulties may have affected these parents’ experiences of the child’s condition and their conversations with the child. These children were included to reflect the true variation in children with craniofacial conditions and also to explore these parents’ experiences. Future research should facilitate further exploration of this vulnerable subgroup to ensure clinical care is inclusive of any additional challenges, particularly given the underrepresentation of children with co-morbidities in the literature (Bates et al., 2020). Another limitation could be that some of the children were not old enough to have conversations about appearance with their parents. Hence, parents of very young children shared their reflections about future appearance-related conversations rather than their actual experiences.

## Conclusion

The findings of this in-depth qualitative study demonstrate the contrast between parents’ own internal adjustment to their child’s different appearance and the impact of external reminders, such as appearance-altering surgeries and social encounters. Parents struggled to decipher whether to raise appearance-related issues with their child and how and when to do this without causing their child distress. Psychological support to help parents cope with difficult feelings around their child’s appearance is indicated, as is guidance regarding how to initiate positive and age-appropriate conversations with their child about appearance.

## Supplemental Material

sj-pdf-1-qhr-10.1177_10497323211039205 – Supplemental material for “Will You Still Feel Beautiful When You Find Out You Are Different?”: Parents’ Experiences, Reflections, and Appearance-Focused Conversations About Their Child’s Visible Difference Supplemental material, sj-pdf-1-qhr-10.1177_10497323211039205 for “Will You Still Feel Beautiful When You Find Out You Are Different?”: Parents’ Experiences, Reflections, and Appearance-Focused Conversations About Their Child’s Visible Difference by Kristin J. Billaud Feragen, Anita Myhre and Nicola Marie Stock in Qualitative Health Research
