# Supplementary material for: “Will You Still Feel Beautiful When You Find Out You Are Different?”: Parents’ Experiences, Reflections, and Appearance-Focused Conversations About Their Child’s Visible Difference
Source: Qual Health Res. 2021 Oct 1;32(1):3–15. doi: 10.1177/10497323211039205 (PMC8739583; doi:10.1177/10497323211039205)
Supplement: sj-pdf-1-qhr-10.1177_10497323211039205 – Supplemental material for “Will You Still Feel Beautiful When You Find Out You Are Different?”: Parents’ Experiences, Reflections, and Appearance-Focused Conversations About Their Child’s Visible Difference [file sj-pdf-1-qhr-10.1177_10497323211039205.pdf]

Supplemental file

Table 1. Semistructured Interview Guide: Questions related to appearance issues

Interview Section	Exemplar Interview questions
Parental perception of appearance	▪ Do you believe other people notice that your son/daughter was born with (diagnosis)?<br>▪ If yes, how do you feel about other people noticing?<br>▪ How do you feel about your child's appearance?
Parental perception of child experience	▪ How do you think your child feels about other people noticing?<br>▪ How do you believe your child feels about his/her appearance?<br>▪ Has your child experienced others commenting or asking about (condition)? If yes, how have you experienced this? How did you cope with this?<br>▪ Has your son/daughter experienced appearance-related teasing? If yes, how have you experienced this? How did you cope with this?
Social experiences	▪ (If reactions from others have been mentioned): How do you cope with other people's reactions to the difference?
Talking about appearance	▪ Do you talk to your child about appearance-related issues? If yes, how do you feel about talking about appearance with your child?
Appearance-altering surgery	▪ Has your child been through operations that have changed his/her appearance? If yes, in which ways? How did you feel about this?<br>▪ How do you believe your child experiences his/her appearance after the operation(s)?<br>▪ How did you experience the child's appearance after operations?
